# Gap-junction channels inhibit transverse propagation in cardiac muscle

**DOI:** 10.1186/1475-925X-4-7

**Published:** 2005-01-28

**Authors:** Nicholas Sperelakis, Lakshminarayanan Ramasamy

**Affiliations:** 1 Dept. of Molecular & Cellular Physiology, University of Cincinnati College of Medicine, Cincinnati, OH 45267-0576, USA; 2 Dept. of Electrical Computer Engineering and Computer Science, University of Cincinnati, College of Engineering Cincinnati, OH 45221, USA

**Keywords:** Transverse propagation in cardiac muscle, Transverse spread of excitation, PSpice simulations, Gap junctions, Junctional cleft potential

## Abstract

The effect of adding many gap-junctions (g-j) channels between contiguous cells in a linear chain on transverse propagation between parallel chains was examined in a 5 × 5 model (5 parallel chains of 5 cells each) for cardiac muscle. The action potential upstrokes were simulated using the PSpice program for circuit analysis. Either a single cell was stimulated (cell A1) or the entire chain was stimulated simultaneously (A-chain). Transverse velocity was calculated from the total propagation time (TPT) from when the first AP crossed a V_m _of -20 mV and the last AP crossed -20 mV. The number of g-j channels per junction was varied from zero to 100, 1,000 and 10,000 (R_gj _of ∞, 100 MΩ, 10 MΩ, 1.0 MΩ, respectively). The longitudinal resistance of the interstitial fluid (ISF) space between the parallel chains (R_ol2_) was varied between 200 KΩ (standard value) and 1.0, 5.0, and 10 MΩ. The higher the R_ol2 _value, the tighter the packing of the chains. It was found that adding many g-j channels inhibited transverse propagation by blocking activation of all 5 chains, unless R_ol2 _was greatly increased above the standard value of 200 KΩ. This was true for either method of stimulation. This was explained by, when there is strong longitudinal coupling between all 5 cells of a chain awaiting excitation, there must be more transfer energy (i.e., more current) to simultaneously excite all 5 cells of a chain.

## Introduction

We have developed an electric field hypothesis for the mechanism of transmission of excitation from one cell to the next that does not require gap-junction channels [1-4]. In the electric field hypothesis, the electrical voltage that develops in the narrow junctional cleft (V_jc_) when the prejunctional membrane generates an action potential, serves to depolarize the postjunctional membrane its threshold, by a patch-clamp-like effect. The parameters that affect the magnitude of Vjc include the size of R_jc_, the transverse resistance of the junctional cleft. This results in excitation of the postjunctional cell, after a brief junctional delay. The total propagation time consists primarily of the summed junctional delays. This results in a staircase-shaped propagation, the surface sarcolemma of each cell firing almost simultaneously [2].

There are no low-resistance connections between the cells in several different cardiac muscle and smooth muscle preparations (reviewed in [3,4]. Propagation by mechanisms not requiring low-resistance connections have also been proposed by others [5-8]. Propagation has been demonstrated to be discontinuous (or saltatory) in cardiac muscle [9-12]. Fast Na^+ ^channels are localized in the junctional membranes of the intercalated discs of cardiac muscle [13-15] Sperelakis, 1995, a requirement for the EF mechanism to work [3,4,1,2,13]. In connexin-43 and Cx40 knockout mice, propagation in the heart still occurs, but it is slowed [15-19], as predicted by our PSpice simulation study [20]. It was reported that the anisotropic conduction velocity observed in the heart is not a result of cell geometry [21].

Subsequently, we published a series of papers on the longitudinal and transverse propagation of action potentials in cardiac muscle and smooth muscle using PSpice analysis [22,20,24]. In the review process for our recent paper [24], one of the referees asked us to determine the effect of introducing strong cell coupling via gap-junction (g-j) channels between cells within each chain on the transverse propagation in our 5 × 5 model (5 parallel chains of 5 cells each). Unexpectedly, we found that strong cell coupling (10,000 or 1,000 g-j channels per junction) actually *inhibited *transverse propagation. This fact was briefly mentioned as an unpublished observation in that paper. The purpose of the present study was to do a thorough investigation of that strange phenomenon. The results showed that, in cardiac muscle, transverse propagation was inhibited when many g-j channels were added between cells of each chain. This was true when either a single cell in the first chain was stimulated (cell A1) or the entire chain (A-chain) was stimulated simultaneously.

## Methods

**Figure 1:**
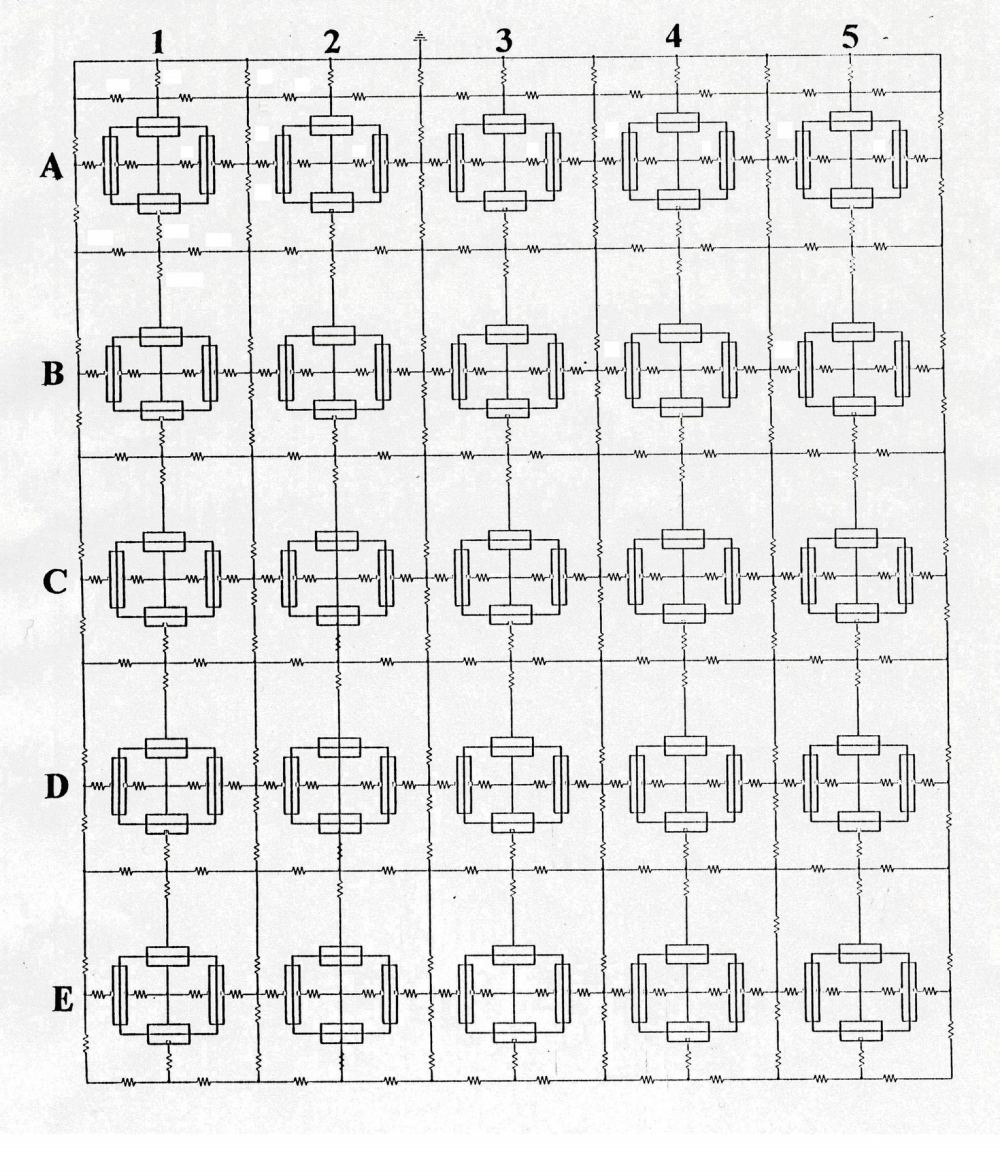
The 5 × 5 model for cardiac muscle, consisting of 5 parallel strands (A-E) of 5 cells each (1–5) (total of 25 cells). Each muscle cell was represented by a block of 4 basic units: 2 units representing the surface membrane (one upward-facing and one downward-facing) and one unit for each of the two junctional membranes. For simplicity, the lumped resistance of the gap-junctions is not indicated here, but is shown in Fig. 2. Transverse propagation is sequential activation of chains A to B to C to D to E.

**Figure 2:**
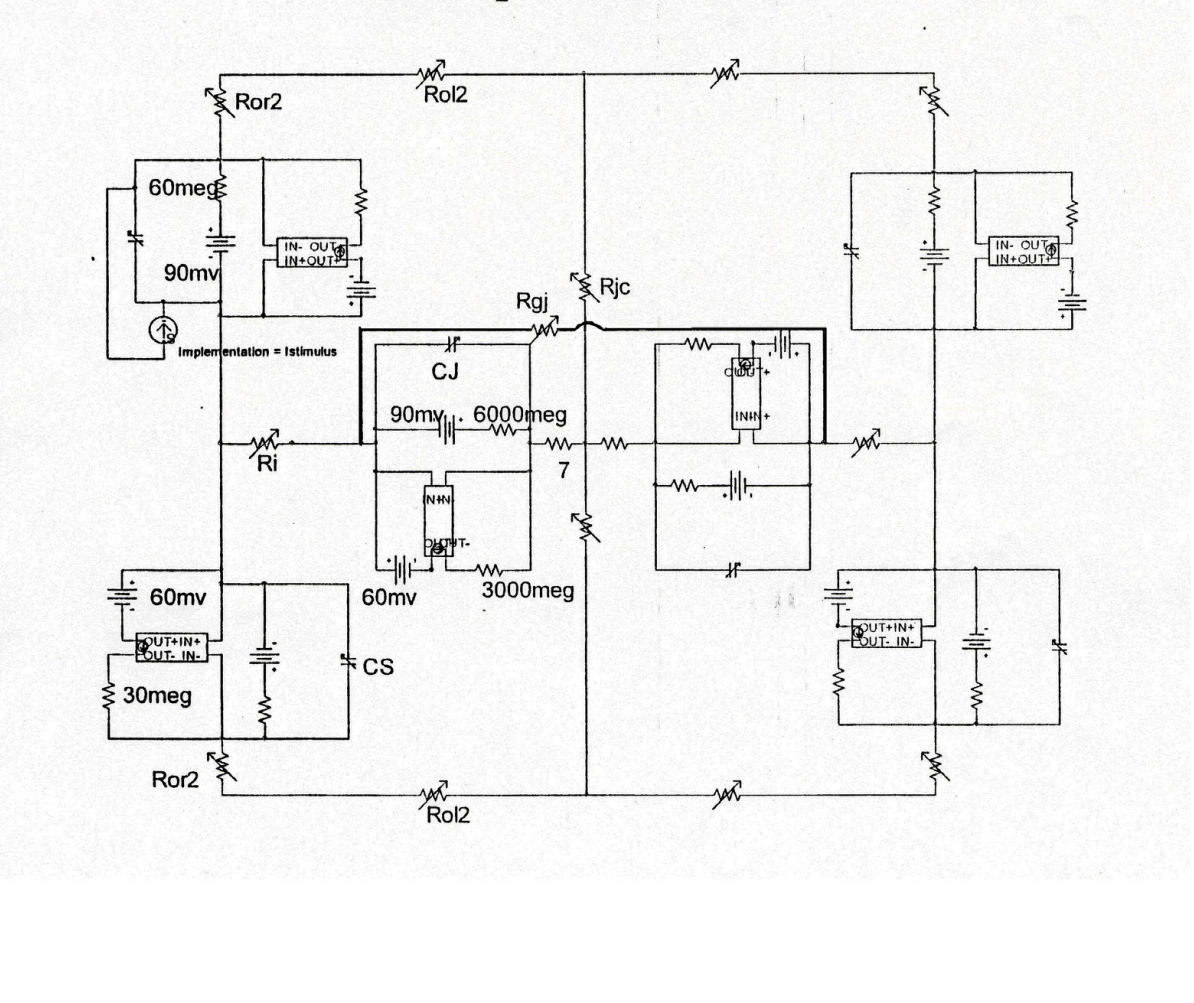
Blow-up of a small portion of the 5 × 5 model to show the electrical circuit for each basic unit, including the "black-box" required for excitability. R_ol2 _represents the longitudinal resistance of the interstitial fluid between the parallel chains; the higher the resistance, the tighter the packing of the chains. Depolarizing current (0.25 nA) is applied to the interior of either the first cell or the entire chain (A-chain) simultaneously. When gap-junction channels were added, a resistor (R_gj_) was inserted across each cell junction, from the interior of one cell to the interior of the next one.

Details of the methods used and modeling of the PSpice analysis were given in our previous papers, including the limitations [21-24]. The full version of the PSpice software for circuit analysis/design was obtained from the Cadence Co. (Portland, OR). The assumptions made were given previously, including the entire circuit that was used [22]. An abbreviated version of the circuitry is given in the first two figures. The surface membrane of each myocardial cell was represented by 2 units and each junctional membrane by 1 unit (Figs. 1, 2). The values for the circuit parameters used under standard conditions were given previously for both the surface units and junctional units in cardiac muscle [22]. The myocardial cell was assumed to be a cylinder 150 μm long and 16 μm in diameter. The myocardial cell capacitance was assumed to be 100 pF, and the input resistance to be 20 MΩ. A junctional tortuosity (interdigitation) factor of 4 was assumed for the cell junction.

The circuit used for each unit was kept as simple as possible, using only those ion channels that set the resting potential (RP) and predominate during the rising phase of the AP. We wanted to only inscribe the rising phase of the APs to study propagation in the 2-dimensional sheet of myocardial cells. It was not possible to insert a second or third "black-box" into the basic excitable units, because the system became erratic, therefore the dynamic behavior of the cardiac cell membrane was an approximation. The RP was -80 mV, and the overshoot potential was +30 mV (AP amplitude of 110 mV). Propagation velocity was calculated from the measured total propagation time (TPT) (measured as the difference between when the APs of the first cell and last cell crossed -20 mV) and cell length.

Because the PSpice program does not have a V-dependent resistance to represent the increase in conductance for Na^+ ^ions in myocardial cells during depolarization and excitation, this function had to be simulated by a V-controlled current source (our "black-box") in each of the basic circuit units (Fig. 2). The current outputs of the black-box, at various membrane voltages, were calculated assuming a sigmoidal relationship between membrane voltage and resistance between -60 mV and -30 mV. The V values used in the GTABLE were those recorded *directly across *the membrane. The excitabilities of the basic units were the same as in our previous papers [21-24].

The upper chain of cells was assumed to be bathed in a large volume of Ringer solution connected to ground. The external resistance (R_o_) of this fluid was divided into two components: a radial (or transverse) resistance (R_or_) and a longitudinal resistance (R_ol_). The longitudinal resistance values (R_ol2_) between the upper chain and interior chains were increased over a wide range to reflect packing of parallel chains into a bundle of fibers, with different degrees of tightness of the packing (Fig. 1). The higher the R_ol2 _value, the tighter the packing of the chains, i.e., the lower the cross-sectional area of the ISF space. The applicable equation is R_ol2 _= ρ (Ω-cm) × L (cm) /Ax (cm^2^), where ρ is the resistivity of the ISF, L is the length, and A_x _is the cross-sectional area of the ISF space. The transverse resistance of the interstitial fluid (ISF) space (R_or2_) also reflects the closeness between the chains; the lower the R_or2 _value, the closer the chains are packed. In the present 5 × 5 model, there were five parallel chains (chains A, B, C, D, and E) of five cells each (cells 1–5). Electrical stimulation (rectangular current pulses of 0.25 nA and 0.50 ms duration) was applied to the inside of either the first cell of chain A (cell A1) or all 5 cells of the A-chain. Under initial conditions, the cells in each chain were not interconnected by low-resistance pathways (gap junction channels), so that transmission of excitation from one cell to the next had to be by the EF developed in the narrow junctional cleft. Then gj-channels were added (100, 1,000 and 10,000 channels per junction) to determine what effect they would have on transverse propagation. Since the number of functional gj-channels per junction have been estimated to be about a 1000 [12], we varied the number over a very wide range. The resistances of the gap-junction channels were lumped into one equivalent resistances because they are all in parallel.

As shown in Figure 1, there were two surface membrane units in each cell (one facing upwards and one inverted) and one unit for each of the junctional membranes (intercalated disks of cardiac muscle). To improve clarity, in some runs the V-recording markers were placed on only one chain at a time. When all cells in a model were being recorded simultaneously (25 cells), the V markers were removed from most of the basic units to minimize confusion. That is, the voltage was recorded from only one surface unit (upward-facing) in each cell. The junctional cleft potential (V_jc_) was recorded across R_jc_, the radial (or transverse) resistance of the narrow and tortuous junctional cleft. Under standard conditions, R_ol2 _was 200 KΩ, R_or2 _was 100 Ω, and R_jc _and was 25 MΩ (50 MΩ ÷ 2).

## Results

The 5 × 5 model (5 parallel chains of 5 cells each) of cardiac muscle was used to examine whether addition of gap-junction (gj) channels between the cells in each chain would affect transverse propagation of simulated (PSpice) action potentials. The number of gj-channels was increased from zero (standard conditions; resistance of the gap junction (R_gj_) of infinite) to 100 (R_gj _= 100 MΩ), 1000 (R_gj _= 10 MΩ), and 10,000 (R_gj _= 1.0 MΩ). Experiments were done with electrical stimulation (0.5 nA, 0.5 ms) of only the first cell of the first chain (A; cell A1) and with simultaneous stimulation of all 5 cells of the A-Chain (each cell receiving 0.5 nA current). This second method of stimulation was done to obtain a more accurate assessment of strictly transverse propagation.

**Figure 3:**
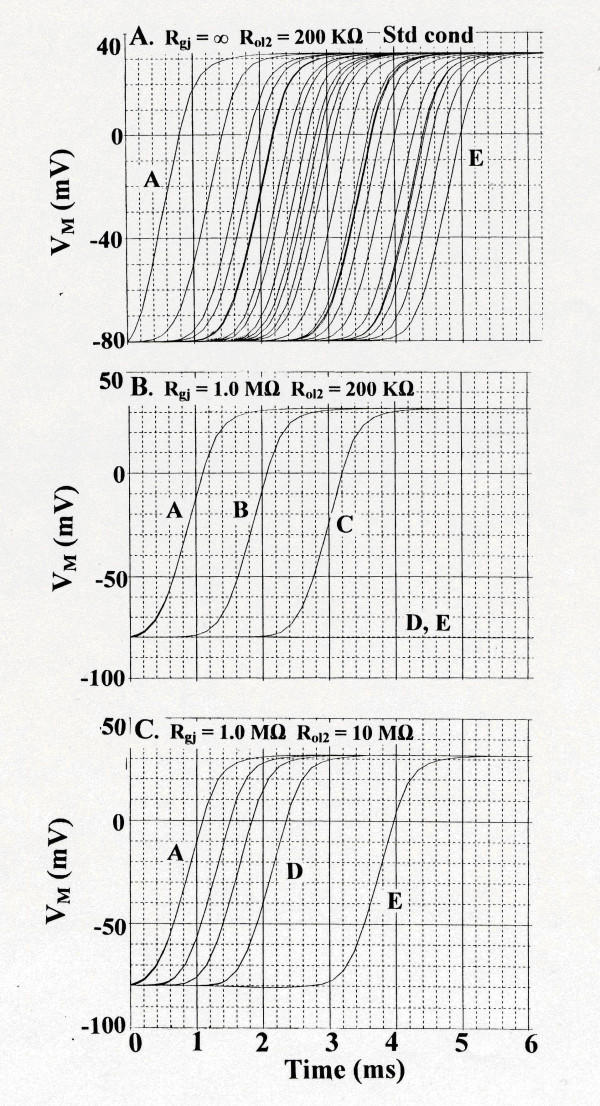
Transverse propagation of simulated action potentials (APs; rising phase) for cardiac muscle (5 × 5 models) with stimulation of one cell only (cell A1; first cell of the A-chain). **A**: R_gj _= ∞ (0 channels). R_ol2 _= 200 KΩ. Standard conditions. All 25 cells responded. **B: **R_gj _= 1.0 MΩ (10,000 channels). R_ol2 _kept unchanged. The last 2 chains (D, E) failed to respond. All 5 cells of each chain that responded (A, B, C) fired simultaneously because of the strong cell coupling. **C: **With R_gj _held at 1.0 MΩ, raising R_ol2 _to 10 MΩ (representing tighter packing of the parallel chains) now allowed all 5 chains to respond. Thus, adding gj-channels inhibited transverse propagation, but this inhibition could be overcome by raising R_ol2_.

Figure 3 illustrates some of the results with stimulation of only cell A1. **Panel A **shows the standard conditions: R_gj _= ∞ (0 gj-channels) and R_ol2 _of 200 KΩ (the longitudinal resistance of the interstitial space between the chains). As shown, all 5 chains (25 cells) responded. The total propagation time (TPT) was 4.2 ms (measured as the elapsed time between when the first and last APs crossed -20 mV). When 10,000 g-j channels were inserted into the cells of each chain (R_gj _= 1.0 MΩ), then the last 2 chains (D and E) failed to respond (**panel B**). The 5 cells of each chain (A, B, and C) that responded now fired simultaneously because of the high degree of cell coupling, thereby giving only three AP traces, as shown. However, raising R_ol2 _to 10 MΩ allowed all 5 chains to respond (**Panel C**), and propagation velocity was increased. These data are summarized in Table 1, **part A**. Hence greatly elevating R_ol2 _could overcome the impaired transverse propagation caused by the gj-channels.

**Table 1:** Summary of simulation data on cardiac muscle with stimulation either of single cell or entire chain.

R_gj _(MΩ)	R_ol2 _(MΩ)	TPT_5c _(ms)	TPT_4c _(ms)	TPT_3c _(ms)	TPT_2G _(ms)	# of chains responding
						
∞	0.2	4.2	3.5	2.7		5
100	0.2	2.4	2.3	2.2		5
10	0.2	----	----	2.1		3
1.0	0.2	----	----	2.1		3
						
∞	1.0	3.8	3.2	2.6		5
100	1.0	3.6	3.1	1.9		5
10	1.0	----	----	1.6		3
1.0	1.0	----	----	1.5		3
						
∞	5.0	2.7	2.6	2.3		5
100	5.0	2.0	1.6	1.2		5
10	5.0	----	----	1.0		3
1.0	5.0	----	----	0.9		3
						
∞	10	2.4	2.2	2.0		5
100	10	1.5	1.3	1.0		5
10	10	2.6	1.2	0.8		5
1.0	10	2.9	1.3	0.8		5
						
∞	0.2	3.6	2.7	2.0	1.2	5
100	0.2	2.5	2.4	2.4	1.2	5
10	0.2	----	----	----	1.1	2
1.0	0.2	----	----	----	1.1	2
						
∞	1.0	2.6	2.1	1.5	0.9	5
100	1.0	2.7	2.6	1.6	0.8	5
10	1.0	----	----	1.6	0.8	3
1.0	1.0	----	----	1.6	0.7	3
						
∞	5.0	1.7	1.4	0.9	0.6	5
100	5.0	1.6	1.4	0.9	0.6	5
10	5.0	----	1.3	0.8	0.5	4
1.0	5.0	----	1.3	0.8	0.5	4
						
∞	10	1.4	1.1	0.8	0.6	5
100	10	1.3	1.0	0.8	0.5	5
10	10	1.2	0.9	0.7	0.4	5
1.0	10	1.2	0.9	0.7	0.4	5
						

**Figure 4:**
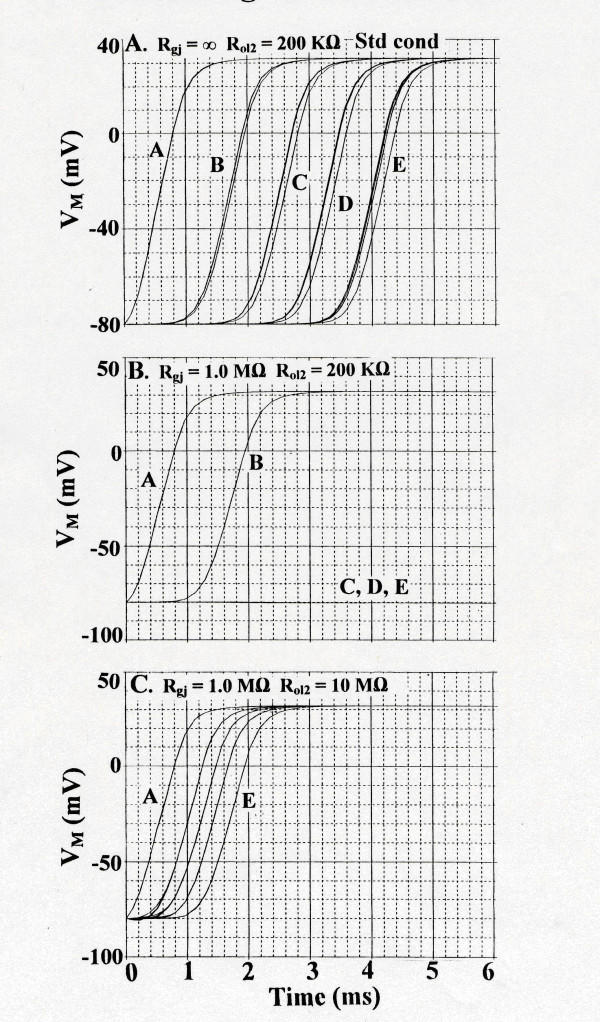
Transverse propagation of simulated APs for cardiac muscle with stimulation simultaneously of the entire A-chain. This was done as a better assessment of transverse propagation for comparison with stimulation of only one cell of the A-chain. **A: **R_gj _= ∞ (0 channels). R_ol2 _= 200 KΩ. Standard conditions. All 5 chains responded. **B: **R_gj _= 1.0 MΩ (10,000 channels). With R_ol2 _kept at 200 KΩ, chains C, D, and E failed to respond. **C**: With R_gj _held at 1.0 MΩ, raising R_ol2 _to 10 MΩ (representing tighter packing of the chains) now allowed all 5 chains to respond. All 5 cells of each chain responded simultaneously because of the strong coupling. Thus, adding gj-channels inhibited transverse propagation, but this inhibition was overcome by raising R_ol2_.

Figure 4 illustrates some of the results with simultaneous stimulation of all 5 cells of the A-chain. **Panel A **shows the standard conditions: R_gj _= ∞ and R_ol2 _= 200 KΩ. As shown, all 5 chains responded (TPT was 3.6 ms). Note that all 5 cells of the B – E chains did not respond simultaneously. When 10,000 gj-channels were added to the contiguous cells of each chain (R_gj _= 1.0 MΩ), then the last 3 chains (C, D, and E) failed to respond (**Panel B**). The 5 cells of each chain that responded (A, B) now fired simultaneously because of the strong coupling. However, elevating R_ol2 _to 10 MΩ allowed all 5 chains to respond (**Panel C**). These data are summarized in Table 1, part B.

**Figure 5:**
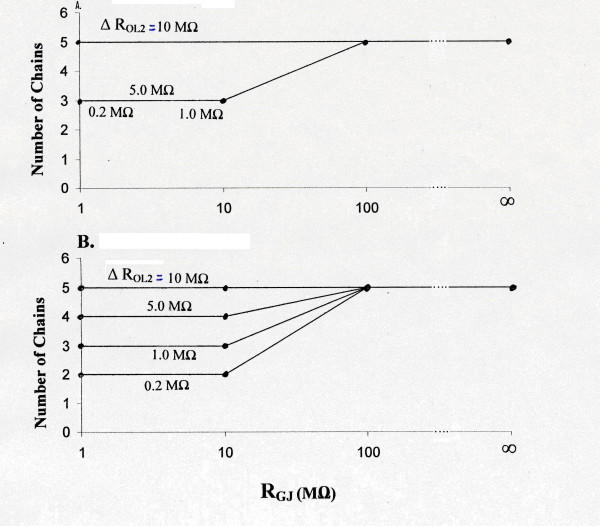
Summary of the transverse propagation experiments. Graphic plot of the number of chains responding as a function of R_gj _for cardiac muscle, with stimulation of only one cell (cell A1) (**panel A**) or with stimulation of the entire A-chain (**panel B**). Data for various R_ol2 _values (0.2, 1.0, 5.0, and 10 MΩ) are indicated. Four different R_gj _values were tested: ∞ (0 channels), 100 MΩ (100 channels), 10 MΩ (1,000 channels), and 1.0 MΩ (10,000 channels). Thus, transverse propagation was depressed when there were many gj-channels (1000 or 10,000), but elevation of R_ol2 _could overcome this depression.

Figure 5 is a graphic summary of the results for both stimulation of only one cell (A1) (**Panel A**) and stimulation of the entire A-chain (**Panel B**). The data include R_gj _values and R_ol2 _values not illustrated in Figures 3 and 4. As such, this figure is complementary to Table 1, but Table 1 lists the TPT values as well. As shown, progressive addition of gj- channels reduces the number of chains that respond, with both ways of stimulation, and progressive elevation of R_ol2 _reverses this inhibition.

## Discussion

The results demonstrated that, in a cardiac muscle model, the insertion of many gj- channels, between abutting cells in each longitudinal chain of cells, actually inhibited transverse propagation of excitation between the parallel chains. This was true both for stimulation of only a single cell of the first chain (A-chain; cell A1) and for stimulation simultaneously of the entire 5 cells of the A-chain. The inhibition produced by the gj-channels could be overcome by greatly increasing the value of R_ol2 _(the longitudinal resistance of the narrow interstitial space between the parallel chains), reflecting tighter packing of the chains.

**Figure 6:**
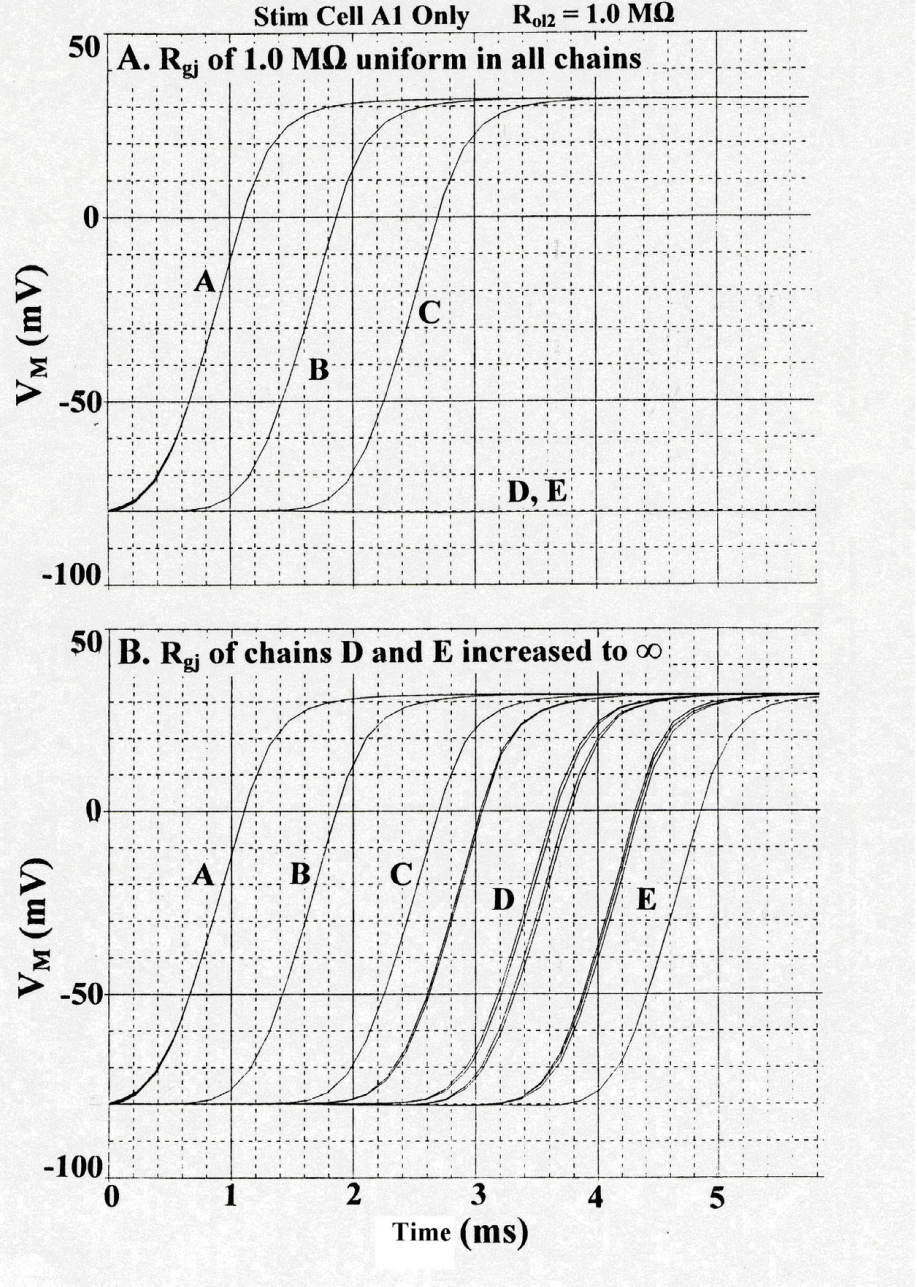
Special experiment to test why presence of many gap-junction channels inhibits transverse propagation. Stimulated Cell A1 only. R_ol2 _of 1.0 MΩ. **A: **Uniform value for R_gj _of 1.0 MΩ (10,000 channels) in all 5 chains. Chains D and E failed to respond. Compare with Fig. 3B for the standard R_ol2 _of 0.2 MΩ. **B: **The R_gj _values in chains D and E only were changed to infinity (0 channels). Now chains D and E responded. See text for details. This demonstrates that removing the gj-channels in the two chains awaiting excitation (D, E) increased the safety factor for transverse propagation.

This finding surprised us at first. We were expecting either that there would be no effect on transverse transmission of excitation or that transverse transfer of excitation would be enhanced. But on further refection, the inhibition produced might be predicted. This is based on the fact that, with strong longitudinal coupling between cells, the entire chain of 5 cells must be simultaneously stimulated to threshold. Therefore, if the transverse transfer energy available is limited and is near threshold for a give chain, it is likely that some chains will fail to fire. Thus, the problem of strong coupling is not in the chains that are already excited, but rather in the quiescent adjacent chain which is in process of trying to become excited (D-chain in case of Fig. 3 and C-chain in Fig. 4). Strong coupling in the "in-process" chain requires more energy transfer from the "already-activated" chain. If the "in-process" chain were not coupled by gj-channels, then if only one cell of the chain received enough stimulating energy to become activated, excitation would spread from it to the remaining cells of that chain. This idea was tested, and the results shown in Figure 6 are consistent with the mechanism proposed above. In **panel A**, in which R_gj _was 1.0 MΩ (10,000 gj-channels) uniformly in all 5 chains, chains D and E failed to fire. When R_gj _of just chains D and E were changed to infinity (0 channels), then all 5 chains fired (**panel B**). Therefore, the inhibition of transverse propagation can be reversed by uncoupling the cells of the chains that failed.

Consistent with the argument presented in the paragraph above, the transverse propagation velocity was not inhibited by insertion of gj-channels, up through the last chain activated (C-chain in case of Fig. 3 and B-chain in Fig. 4). If anything, the transverse velocity was increased slightly (TPT lowered). The TPT values (for differing amount of transverse spread of excitation, e.g. 2-chains, 3-chains, 4-chains, or all 5-chains) are listed in Table 1 for different degrees of cell coupling. Note that, in part A, the largest change in TPT value is between R_gj _of ∞ (0 channels) and R_gj _of 100 MΩ (100 channels); adding more channels (Rgj of 10 MΩ (1000 channels) or 1.0 MΩ (10,000 channels)) did not further decrease TPT. Similar results were found when the entire A-chain was simulated (part B of Table 1).

The reader should be alerted to the limitations of the PSpice program. It was not possible to insert a second or third "black-box" in the basic excitable units (because the system went berserk). Therefore, the dynamic behavior of the cardiac cell membrane was only a close approximation.

We previously found (unpublished observation) that the transverse velocity was greater when size of the model was increased (eg, 3 × 4, 5 × 5, 7 × 7) up to a presumed maximum, indicating that the boundary conditions affected behavior of the model. Therefore, the present experiments should be repeated on larger-sized models. But we believe that the qualitative findings would be much the same. In addition, 2-dimensional activation maps should be made in the future to better elucidate how the wavefront spreads.

These findings might have important clinical implications, especially for the genesis of arrhythmias in pathophysiological situations. Any pathology that altered the number of functioning gj-channels, not only would affect longitudinal velocity of propagation, but also the transverse propagation ability and velocity. Therefore, the genesis of some arrhythmias, e.g., the reentrant type, could be promoted under such conditions.
